# Evaluation of Cytotoxicity and Antibacterial Activity of a New Class of Silver Citrate-Based Compounds as Endodontic Irrigants

**DOI:** 10.3390/ma13215019

**Published:** 2020-11-06

**Authors:** Luigi Generali, Carlo Bertoldi, Alessandro Bidossi, Clara Cassinelli, Marco Morra, Massimo Del Fabbro, Paolo Savadori, Nidambur Vasudev Ballal, Luciano Giardino

**Affiliations:** 1 Department of Surgery, Medicine, Dentistry and Morphological Sciences with Transplant Surgery, Oncology and Regenerative Medicine Relevance (C.H.I.M.O.M.O.), University of Modena and Reggio Emilia, 41124 Modena, Italy; 2 Laboratory of Clinical Chemistry and Microbiology, I.R.C.C.S. Orthopedic Institute Galeazzi, 20161 Milan, Italy; alessandro.bidossi@grupposandonato.it; 3 Nobil Bio Ricerche srl, Via Valcastellana 26, 14037 Portacomaro, Italy; ccassinelli@nobilbio.it (C.C.); mmorra@nobilbio.it (M.M.); 4 Department of Biomedical, Surgical and Dental Sciences, University of Milano, 20122 Milan, Italy; massimo.delfabbro@unimi.it; 5 I.R.C.C.S. Orthopedic Institute Galeazzi, 20161 Milan, Italy; paolo_savadori@yahoo.it; 6 Department of Conservative Dentistry and Endodontics, Manipal College of Dental Sciences, Manipal, Manipal Academy of Higher Education, 576104 Karnataka, India; drballal@yahoo.com; 7 Freelance Researcher, 88900 Crotone, Italy

**Keywords:** biofilm, confocal laser scanning microscopy, endodontic irrigant, *Enterococcus faecalis*, M.T.T. assay, silver citrate

## Abstract

In the present study, the cytotoxicity and the antimicrobial activity of two silver citrate-based irrigant solutions were investigated. Cytotoxicity of various concentrations (0.25%, 0.5%, 1%, 2.5%, 5%) of both solutions (BioAKT and BioAKT Endo) was assessed on L-929 mouse fibroblasts using the MTT assay. For the quantitative analysis of components, an infrared (I.R.) spectroscopy was performed. The minimum inhibitory and minimal bactericidal concentrations (M.I.C. and M.B.C., respectively) were ascertained on *Enterococcus faecalis* strain ATCC 4083. For biofilm susceptibility after treatment with the irrigating agent, a minimum biofilm eradication concentration (M.B.E.C.) and confocal laser scanning microscope (C.L.S.M.) assays were performed. Quantification of *E. faecalis* cell biomass and percentage of live and dead cells in the biomass was appraised. Normality of data was analyzed using the D’Agostino & Pearson’s test and the Shapiro–Wilk test. Statistical analysis was performed using one-way analysis of variance (ANOVA) and Tukey’s test. Both silver citrate solutions showed mouse fibroblasts viability >70% when diluted to 0.25% and 0.5%. Conversely, at higher concentrations, they were extremely cytotoxic. F.T.-IR spectroscopy measurements of both liquids showed the same spectra, indicating similar chemical characteristics. No substantial contrast in antimicrobial activity was observed among the two silver citrate solutions by using broth microdilution methods, biofilm susceptibility (MBEC-HTP device), and biomass screening using confocal laser scanning microscopy (C.L.S.M.) technique. Both solutions, used as root canal irrigants, exhibited significant antimicrobial activity and low cytocompatibility at dilutions greater than 0.5%.

## 1. Introduction

Optimal disinfection of the root canal and prevention of reinfection post-treatment is the primary goal of endodontic therapy [1]. Previous literature scrutinizing debridement and remnant microbes in root canal post-cleaning and shaping protocol revealed deficient debridement [2] and incomplete disinfection of the root canal system [3]. At present, there is no single irrigant that can dissolve organic tissue, destroy bacteria, and demineralize the smear layer concurrently [4]. Hence, it is necessary to combine the use of an oxidizing agent to dissolve necrotic tissue and a chelating agent as a final rinse to eradicate the smear layer. To overcome this limit, sodium hypochlorite (NaOCl), followed by ethylenediaminetetraacetic acid (EDTA), as a final rinse, is the most preferred irrigation protocol employed by clinicians during endodontic treatment [5]. NaOCl alone is incapable of eradicating the mineral content of the smear layer formed on the canal walls during biomechanical preparation [6]. Chelators like EDTA or citric acid (C.A.) are then needed to eliminate the inorganic constituents of the smear layer [7]. Owing to the calcium ion chelating property and elimination of mineral content of the smear layer, EDTA is widely proposed and utilized [8,9]. Though as chelator, EDTA has been popular among clinicians, its drawback lies with its insubstantial antimicrobial efficacy [10]. Limited smear layer elimination in poorly penetrable areas of the root canal can be attributed to its high surface tension value [11].

Regarding its biocompatibility, Nygaard-Ostby [8] showed that there was no sign of periapical tissue damage after 14 months, even though EDTA was forcefully extruded through the apical foramen. In contrast, clinical studies have revealed that EDTA extrusion through the apical constriction can cause irreversible decalcification of the periapical bone and affect the neuroimmunological regulatory mechanisms (inflammatory reactions and immune response) involved in the periapical lesion [12]. Other studies conducted by cytotoxicity assay on cell cultures confirmed the cytotoxic effects of EDTA at various concentrations [13,14]. A recent study, using cell viability 3-(4,5-dimethylthiazol-2-yl)-2,5-diphenyltetrazolium bromide (M.T.T.) assay and genotoxicity, showed that EDTA had the lowest cytotoxic potential among the other irrigants, used either individually or in combination [15], which contradicts the previous literature.

Citric acid (C.A.), a weak organic acid, has been recommended as a substitute to EDTA to eliminate the smear layer [16]. Due to its low pH, C.A. causes more dentine erosion than EDTA and other chelators [17] and leaves precipitated crystals on the root canal wall that might interfere with the root canal filling [18]. However, it has some advantages, as it is more biocompatible and less cytotoxic than EDTA. Its antimicrobial activity has been demonstrated against planktonic bacteria but not against *Enterococcus faecalis* biofilms [18]. Some studies have shown that, although C.A. is less cytotoxic in comparison to other chelating agents [19,20], it has short and long term damaging effects on in vitro cell cultures, causes a decalcifying action on periapical bone, and affects inflammatory and neuro-immune regulation when extruded into periradicular tissues [19,20,21]. The detrimental effect on vital cells was associated with its acidic pH [22]. Since there is, to date, no ideal irrigation solution, alternative solutions are continually being researched and proposed.

In a recent study [23], a disinfectant for surfaces, based on a patented blend of electrolytically generated silver ions (0.003%) in citric acid (4.846%) (BioAKT, New Tech Solutions s.r.l., Brescia, Italy), has been tested as an innovative biomaterial for root canal cleaning and disinfecting. Due to its antibacterial properties, silver and its compounds have been in use for centuries and recently became available as a disinfectant for surfaces and medical instruments and devices [24]. Silver must be in an ionic form to effectively kill microorganisms, as previously established [24]. This patented aqueous disinfectant is an antimicrobial agent based on a stabilized silver ion complex produced by a unique electrochemical process with silver and citric acid, wherein a silver ion is weakly bonded to a citrate ion developing the molecular complex AgC_6_H_7_O_7_ [25]. This disinfectant agent, thus constituted, therefore provides a stabilized form of silver ion in an organic acid (citric acid). The bioavailability of the ions allows the silver citrate complex to be rapidly efficacious against a broad spectrum of bacteria, viruses, and fungi [26,27]. Liau et al. [28] confirmed silver ions affinity for thiol-containing groups. Indeed, silver ions are highly attracted by sulfur-containing thiol groups found in metabolic and structural proteins bound to the bacterial membrane surface. Silver citrate targets these structural proteins and dismantles their structure, leading to the disruption of the organism’s membrane and subsequent lysis of the microbial cells.

Biocompatibility is an important aspect that must be carefully evaluated before a chemical is released to the market [29]. According to Peters [29], it is essential to note that specific international standards exist in this area (ISO 10993 series, especially 10993-5 and 10993-10), but researchers often fail to consider these standards. Recently, based on the promising results obtained previously [23], another silver citrate solution (BioAKT Endo, New Tech Solutions s.r.l., Brescia, Italy) has been introduced for clinical usage to create a new two-in-one endodontic solution. This novel irrigant was reported as non-toxic and biocompatible [30] but there are no reports available to date in the literature about its biocompatibility and composition. Due to the lack of knowledge on this class of compounds in the literature, this study aimed to assess the cytotoxicity and the antimicrobial activity of BioAKT Endo and compare its chemical composition, pH, and antibacterial activity to BioAKT disinfectant.

## 2. Materials and Methods

The pH of these solutions was calculated using a pH meter G.L.P. 22 (Crison Strumenti S.p.A., Carpi, Italy), obtaining a value of 1.7 for both chemicals.

### 2.1. Cytotoxicity Test

The toxicity of serial dilutions of BioAKT Endo and BioAKT was measured in vitro, according to ISO 10993-5:2009 standard [31]. The L-929 mouse fibroblasts cell line (BS CL 56) was obtained from Istituto Zooprofilattico Sperimentale della Lombardia e dell’ Emilia (I.Z.S.L.E.R.), Brescia, Italy. Cells were cultivated in Minimum Essential Medium (M.E.M. + GlutaMAX, Thermo Fisher Scientific, Rodano, Italy) supplemented with 10% fetal bovine serum (Gibco, Life Technologies Srl, San Giuliano Milanese, Italy), L-glutamine 2 mM (Gibco, Life Technologies Srl, San Giuliano Milanese, Italy), 100 IU/mL penicillin (Gibco, Life Technologies Srl, San Giuliano Milanese, Italy), 100 µg/mL streptomycin (Gibco, Life Technologies Srl, San Giuliano Milanese, Italy), and 0.25 µg/mL amphotericin B (Gibco, Life Technologies Srl, San Giuliano Milanese, Italy). The cells were plated at 25,000 cells cm^−2^ (2.5 × 10^4^, TC10 cell counter, Bio-Rad Laboratories Inc., Hercules, CA, USA) in 12 well plates (Sarstedt S.r.l., Trezzano sul Naviglio, Italy), then placed into an incubator (H.E.R.A. Cell, Thermo Fisher Scientific, Rodano, Italy) at 37 °C in a humidified atmosphere of 5% CO2 for 24 h. At the end of this period, the medium was removed and replaced with a fresh medium containing 100 µL volumes of the diluted test solution (0.25%, 0.5%, 1%, 2.5%, 5%) added to the relevant wells and incubated for 72 h. In addition to the test solutions, a control group (C) that contained cells and culture medium alone with no irrigants was included. Subsequently, the L-929 cells grown in the presence of different concentrations of liquids were observed under an inverted optical microscope (D.M.I. 4000B, Leica, Buccinasco, Italy) to evaluate the presence of dead cells, multinucleated giant cells, and general anomalies of cellular morphology. All observations were compared with the controls. Cell viability in contact with the tested material was evaluated using a proven reliable test, 3-(4,5-dimethylthiazol-2-yl)-2,5-diphenyltetrazolium bromide (M.T.T.) assay, according to the Mosmann method [32]. Briefly, the M.T.T. allows to highlight the presence of toxic effects of the materials thanks to the decrease in the enzymatic activity of the mitochondrial enzyme succinate dehydrogenase (SDH), which reduces, during the three hours of incubation at 37 °C, the initially soluble tetrazolium salts, originally yellow stained, in a blue/purple insoluble salt in water, formazan: the greater the quantity of sediment, the higher the number of viable cells. The medium was removed and instantly substituted with 100 μL/well of 1.0 mg/mL M.T.T. dissolved in the medium (Sigma-Aldrich, Milano, Italy). Afterward, after 3 h of incubation at 37 °C under 5% CO_2_ and 95% humidity, the supernatants were discarded solubilized with dimethyl sulfoxide (DMSO Sigma-Aldrich, Milano, Italy) and measured spectrophotometrically. The solutions were read at 560 nm wavelength using a spectrophotometer SPARK 10M (Tecan Italia Srl, Cernusco Sul Naviglio, Milano, Italy). Each test was performed using three cultures for both solutions and repeated four times.

### 2.2. F.T.-IR Spectroscopy in Attenuated Total Reflectance (A.T.R.)

This method was used to carry out the in situ quantitative analysis of components present in both citric acid-based solutions (Bioakt and BioAKT Endo) to compare whether both solutions differed from each other or not. The infrared (I.R.) spectroscopy measurements were performed by the attenuated total reflectance (A.T.R.) using a Nicolet iS10 FTIR Spectrometer (Thermo Fisher Scientific, Rodano, Italy) equipped with a monolithic diamond A.T.R. crystal iD7 A.T.R. Water is a very strongly absorbing and temperature-dependent material in both the near and mid-infrared (I.R.) spectral regions [33]. As such, water creates considerable background problems for many infrared applications. To overcome these drawbacks, 0.5 mL of the liquid from both chemicals were initially deprived of the aqueous phase by evaporation at 37 °C in a ventilated oven S.T.Z.-N 52 (Falc Instruments Srl, Treviglio, Italy), to create solid compounds that allow infrared radiation to pass through them. After evaporation, the analytes, free from their aqueous background, were recovered and placed on the diamond crystal on which they were pressed with the appropriate accessory. Each spectrum was obtained by performing 32 scans with a resolution of 4 cm^−1^ in the spectra range 500–4000 cm^−1^.

### 2.3. Assessment of Antimicrobial and Antibiofilm Activity

*E. faecalis* strain ATCC 4083 was obtained from American Type Culture Collection, (A.T.C.C. Manassas, VA, U.S.A.) in frozen stock and stored at −80 °C until analysis and herein used because it was primarily isolated from the root canal of the pulpless tooth [34], mimicking a clinical environment. Before application, the strain was liquified and reconstituted in tryptic soy agar (T.S.A., Biomérieux, Marci l’Etoile, France) for 24 h at 37 °C. Minimum inhibitory concentration (M.I.C.) and minimum bactericidal concentration (M.B.C.) of both silver citrate solutions were obtained by using the broth microdilution method, as previously described [35]. Briefly, the resuspended bacterial culture was inoculated to reach a final concentration of 5 × 10^6^ CFU/mL in a 96-wells microplate containing serial 2-fold dilution of the tested solutions. Minimum inhibitory concentration values were interpreted after 24 h of incubation at 37 °C by visually inspecting the turbidity of the wells following C.L.S.I. standard guidelines [36]. The M.B.C., stated as the lowest concentration of an antimicrobial substance able to kill 99.9% of the initial inoculum, was performed by subculturing 10 µl of microbial suspension from wells showing no visible growth in the M.I.C. microdilution tests onto agar plates to count residual cells.

Likewise, M.B.C. values were interpreted after 24 h of incubation at 37 °C. For biofilm susceptibility, the minimum biofilm eradication concentrations (M.B.E.C.) was derived by employing the MBEC-HTP device (Innovotech, Edmonton, Alberta, Canada) as thoroughly described by Giardino et al. [35]. Brain Heart Infusion (B.H.I.) broth inoculated with 10^7^ CFU/mL of *E. faecalis* was distributed in the microplate wells to organize a biofilm on the pegs located on the lid of the device. After 24 h incubation at 37 °C, the pegs were rinsed with sterile saline to remove unattached cells. Subsequently, the lid was put on a ‘challenge plate’ carrying serial 2-fold dilution of the testing solutions for 1 and 3 min while agitating on an orbital shaker and moved again in a new rinse plate for 30 min to neutralize the test solutions. The lid was then placed in a new 96-well microplate containing fresh B.H.I. broth and sonicated to dislodge the remaining biofilm on the pegs. The lid was then detached, restored with a non-pegged lid, and the plate incubated overnight at 37 °C. M.B.E.C. values were obtained by visually inspecting the wells for turbidity [37]. Clear wells suggested a full biofilm eradication. The evaluation of biomass removal and killing activity by the testing solutions was carried out on *E. faecalis* biofilm grown on uncoated 10-mm diameter glass slides for 48 h by inoculating bacterial cells in 1 ml of B.H.I. broth to a final concentration of 10^7^ CFU/mL at 37 °C and treated with both solutions for 1 and 3 min (controls were treated with sterile saline). The quantification was done by using a confocal laser scanning microscopy (C.L.S.M.) TCS SP8 (Leica Microsystems C.M.S. GmbH, Mannheim, Germany) using a 20 × dry objective (HC PL Fluotar 20 × /0.50 DRY according to an established method previously described [35]. Briefly, mature biofilms on glass slides were stained with Filmtracer™ LIVE/DEAD™ Biofilm Viability Kit (Thermo Fisher Diagnostics SpA, Rodano, Italy) after the removal of unattached cells and analyzed by acquiring images from no minimum than three random areas from three replicates. The quantification of cell biomass was expressed in µm^3^. The percentage of live and dead microorganisms in the biomass was evaluated in each group.

To confirm the smear removal ability of BioAKT Endo undiluted and diluted at 0.5% (the undiluted solution not cytocompatible, the second instead cytocompatible as found below), a preliminary study was executed on six teeth (data available as Supplementary Materials), using an established method [35]. The reason why BioAKT Endo was also investigated at a concentration of 0.5% in a few samples was to determine whether it retained its ability to eliminate the smear layer even in its diluted and no cytotoxic formula. Six intact single-rooted human teeth, after the biomechanical preparation of the root canals, were categorized into two groups (three samples each) based on the final rinse utilized. A supplemental tooth served as a positive control (final rinse with distilled water). Subsequently, the specimens were split into two halves, coated with gold, and explored using scanning electron microscopy (S.E.M.) Nova NanoSEM 450 (F.E.I. Company, Eindhoven, The Netherlands) at 3000× and 6000× magnifications. A chemical microanalysis with a dispersive energy X-ray (E.D.X.) was performed using an X-Max50 detector and AZtecEnergy software (Oxford Instruments, Abingdon, Oxfordshire, U.K.) connected to the Nova NanoSEM 450 device to identifying and quantifying the composition of the root dentin surfaces after irrigation.

### 2.4. Statistical Analysis

The normality of data distributions was evaluated using the D’Agostino & Pearson’s test and the Shapiro–Wilk test. Results were displayed for data distributed normally as means and standard deviations (S.D.), plus 95% confidence intervals (CI). For comparisons of viability among different concentrations for each solution, one-way analysis of variance (ANOVA) and Tukey’s test was performed. For between-group viability comparisons at each concentration, the Mann–Whitney test was applied. To analyze the results of biofilm treatment, comparisons between the two groups were performed by employing a two-tailed, unpaired Student’s *t*-test for each strain. *p* = 0.05 value was set like a significance threshold. The software GraphPad Prism 5.0 (GraphPad Software, La Jolla, CA, U.S.A.) was undertaken for statistical analysis.

## 3. Results

Both BioAKT solutions showed mouse fibroblasts viability >70% up to 0.5% concentration, at higher concentrations (1, 2.5, 5%) instead it was extremely cytotoxic. Table 1 displays the results of cell viability in the control and the experimental groups.

The analysis of variance (ANOVA) showed that there was a highly significant within-group difference among mouse fibroblasts viability at the different concentrations (*p* < 0.0001) for both solutions. The results of the Tukey’s multiple comparison test for BioAKT and BioAKT Endo are reported in Table 2 and Table 3, respectively.

Employing the broth microdilution method (M.I.C. and M.B.C.), both solutions maintained the same inhibitory and bactericidal profile against enterococcal planktonic cells, inhibiting cell growth even if diluted 1:16 and killing more than 99.9% of inoculated cells up to a dilution of 1:8 (Table 4).

**Figure 1:**
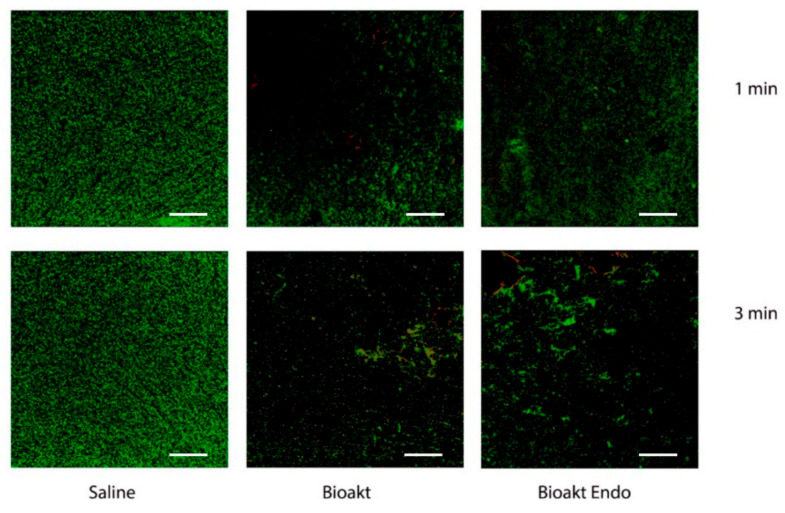
C.L.S.M. 3D images of *E. faecalis* biofilms treated with the tested solutions and control (saline solution). Live cells are seen in green, whereas dead cells are seen in red. Scale bar: 100 µm.

**Figure 2:**
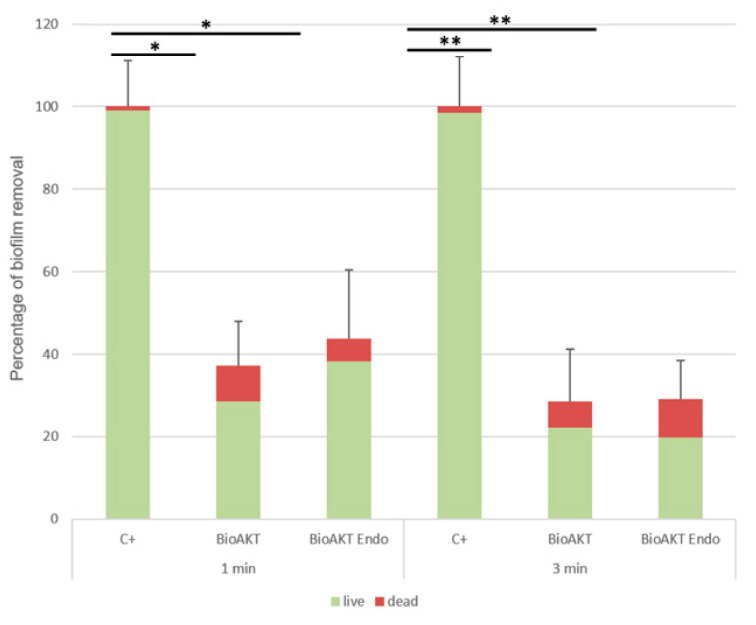
Biofilm removal efficacy of the tested solutions on pre-formed *E. faecalis* biofilm analyzed by C.L.S.M. Residual biomass is represented in percentage upon treatment concerning the control. Whole bars represent the total biomass; the green fraction is viable cells, in red the dead cells still encased in the biofilm. * *p* < 0.05, ** *p* < 0.01.

When evaluating the minimum biofilm eradicating concentration at standard conditions, both chemicals were able to dislodge the biofilms formed on the pegs at dilutions of 1:8 and 1:16, respectively, when treated for 1 min and 3 min (Table 4). Biofilm dispersal treatment was also investigated, employing C.L.S.M. Figure 1 and Figure 2 displayed that both irrigant solutions had a good removal effect on *E. faecalis* mature biofilm, with a statistically significant reduction already at 1 min treatment (*p* < 0.05). The increase in the exposure time only slightly increased the treatment effect compared to the positive control (*p* < 0.01), although not significantly compared to the 1 min treatment.

In the sessile biomass that endured on the glass slide surface, an augmented number of dead cells could be appreciated (Table 5). A significant increase was observed only for 3 min treatment with the BioAKT Endo solution (Table 5).

**Figure 3:**
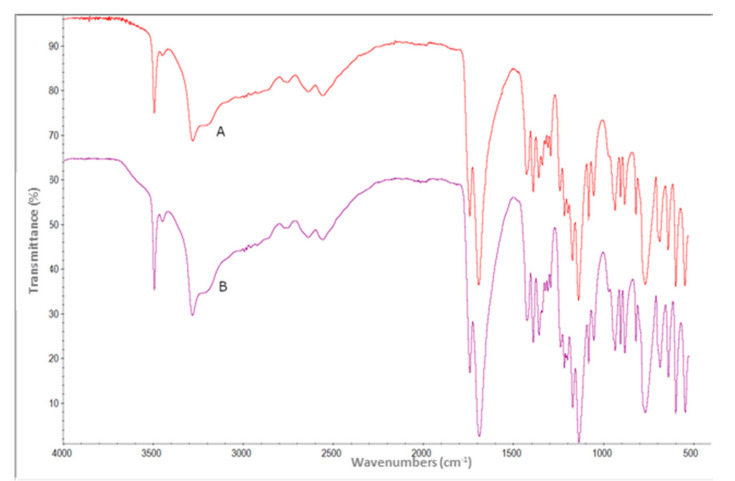
Infrared spectra of BioAKT Endo (**A**) and BioAKT (**B**).

The infrared (I.R.) spectroscopy measurements of both liquids showed the same spectra (Figure 3), suggesting a similar nature of this new class of irrigants. The I.R. spectra obtained were superimposable and highlighted the same characteristics. The observed bands were attributable to the components of the samples examined and had the same features in the two solutions analyzed. Indeed, the emission spectrum is different for every element, acting as an atomic fingerprint by which the elements can be identified. Identical chemical characteristics, pH value, and comparable antibacterial action were found for both solutions investigated in the present study.

Since analysis was performed on residual left after evaporation of the liquid phase, as reported in the Materials and Methods section, it cannot be completely excluded that volatile compounds, possibly different as to nature and amount, were originally contained in the two tested irrigants. This hypothesis seems unlikely however, since no mention of further compounds is made in the relevant literature or in the Material Safety Data Sheets of the irrigants.

Limited to the number of teeth examined, the supplemental scanning electron microscopy (S.E.M.) study based on the final rinse solution used found that the dentinal walls were coated with the smear layer in the control (distilled water), without open dentinal tubules exposed. In contrast, the final rinse with undiluted and diluted 0.5% BioAKT Endo exhibited no smear layers in the coronal and middle third of root canals, instead covered the dentinal wall in the apical third of all the samples, irrespective of the irrigating solution used (undiluted and diluted 0.5%). The SEM-EDX analysis highlighted micro-agglomerations on the root dentin irrigated with undiluted solution principally constituted of silver, calcium, and phosphorous (Figures S1 and S2), contrary to the diluted solution where the presence of silver was not appreciable.

## 4. Discussion

An ideal root canal irrigant should have strong antibacterial properties and minimal cytotoxic effects on the host tissues. Then one must weigh their therapeutic benefits against their potential cytotoxicity effects [38]. Because root canal irrigants can come in contact accidentally with periradicular tissues, in addition to having a useful antibacterial ability, also their biocompatibility should be evaluated [39]. In spite of the widespread use of silver and silver ions in industry and for medicinal purposes, there is only inconsistent information on its toxicity. The superiority of silver, due to its established bactericidal action, must be weighed against possible tissue damage due to its cytotoxic nature [40,41]. Environmental and human studies suggest that some forms of silver, particularly those that dissociate and release free silver ions (Ag^+^), are more toxic than others. Greulich et al. [40] have shown that the effective toxic concentration of silver ions and silver nanoparticles towards bacteria and human cells is almost the same. The same results were previously obtained by testing the same concentration of Ag^+^ ions on human fibroblasts, bacteria, fungi, and algae [41]. Glutathione (G.S.H.) has antioxidant properties effective against injury to cells induced by reactive oxygen species (R.O.S.) such as free radicals, peroxides, lipid peroxides, and heavy metals, maintaining cellular oxidation-reduction homeostasis [42]. Reactive oxygen species (R.O.S.) are continually created and destroyed in biological systems. They play an essential part in various normal biochemical functions, and abnormality in their functions leads to pathological processes [42]. Ag^+^ may influence cellular redox status and give rise to reactive oxygen species (R.O.S.) within mammalian cells, decreasing the concentration of G.S.H. because of oxidative stress.

Recent studies confirmed that Ag^+^ exhausts G.S.H. and causes oxidative stress, which leads to cellular necrosis [43], and clarified that the antibacterial effects of Ag^+^ are related to its role in stimulating R.O.S. generation [44]. Our findings were in line with those presented above. BioAKT Endo, similarly to BioAKT, indeed, has proven high cytotoxicity at a concentration higher than 0.5% (Table 1). Furthermore, it can be speculated that the cytotoxic effect of these irrigants could also be related to their low pH (1.7) because of the presence of citric acid (C.A.) in both novel mixtures. Previously, some reports showed that C.A. could affect the irrigants biocompatibility due to its acidic pH [45,46], confirming our hypothesis. In another study, Lan et al. reported [22] that the detrimental effect on vital cells was associated with its acidic pH for the ability of C.A. to diminish the pH value of the culture medium, causing extracellular acidosis [47]. Unsurprisingly, in this study, at concentrations higher than 0.5%, both tested solutions had a lower cytocompatibility than the control group.

Interestingly, in our preliminary laboratory study, we found that the surface tension value of both C.A.-based chemical agents was halved (32.00 mJ/m^2^) if compared to that of C.A. solution without detergent added (69.00 mJ/m^2^) [48]; this was probably due to the incorporation of a wetting agent into both solutions, according to the original patented formula [49].

The silver ions in an aqueous solution have a limited stable ionic life. After a limited time, they aggregate and form complexes with other elements, thus diminishing the concentration of the silver ions within the aqueous solution. To overcome these problems, detergents were added to the silver citrate complex as a stabilizer in the original formula, improving the stability of the Ag-based suspension [50]. As well as stabilizers, the surface agents, when added to irrigants, reduce their surface tension, enhancing the antibacterial effect of the solutions, and reducing the mechanical stability of the biofilm by destabilizing the cohesive forces of the biofilm [38]. Despite these practical effects, a study highlighted that detergents have cytotoxic activity on prokaryotic cells, suggesting their cautious use. [51]. This indicates that the preferred use of Ag ions and AgNPs is in the field of biocidal or antiseptic agents, but not in biomedicine.

There is no consensus with reference to the ideal concentration of C.A. to use in clinical practice because it has been used at different concentrations, between 1% and 50% [52,53,54,55,56,57]. Some studies showed that using a low concentration of citric acid and low pH is adequate to remove the smear layer [53,54,56].

Furthermore, a lower concentration of citric acid (1%) has been recommended as a useful option for the clinical eradication of the smear layer from dentin walls [55], to elude erosion of root canal dentin [57]. However, although 1% citric acid is as efficacious as EDTA in eliminating the smear layer, the acidic pH may irritate periapical tissues [58]. A statistically significant difference has been reported between 5% citric acid at pH 1.9 and the same solution with a pH buffered to 6.0 [53]. The smear layer remained on the dentinal walls and tubules in the buffered solution, contrary to the 5% citric acid bufferless solution, wherein the smear layer was removed from root canal walls and the dentinal tubules. In the study mentioned above [53], elimination of the smear layer by citric acid solutions with higher concentrations buffered to pH 6 was insufficient, suggesting that the pH of the solution was the decisive factor in eliminating the smear layer rather than the concentration, in line with Hennequin et al. findings [52].

To date, a variety of assays to measure the number of viable cells in culture have been proposed for assessing cell reactions to external factors. Herein, an established method, M.T.T. assay [32], was used because it is currently the most widely employed method of estimating the number of viable cells in multiwell plates. Furthermore, it is a very rapid, sensitive, and precise method to measure the activity of all cell lines [59], and it is the most often cited in the scientific literature as evidenced by thousands of published articles [60] and, last but not least, it is extensively applied by the researchers in cell studies worldwide. It has been highlighted by ISO 10993-10:2010 standard [61] that if the pH of the test sample is ≤2.0 or ≥11.5, the material shall be considered an irritant. Although this assay is recommended to evaluate the cytotoxicity of chemicals intended for endodontic use [29], it is not suitable for animal tests to safeguard their welfare [62]. Due to the acidic pH of both solutions tested, this ISO assay was not employed in the present investigation.

To declare a medical device compliant, it must not be irritating for skin and oral mucosa, more than cytotoxic in vitro assays. A chelating agent, EDTA 17%, also showing high cytotoxicity [63], thanks to its neutral pH, has been proven not irritating for skin and oral mucosa and recommended for clinical use (Chelab Srl-Merieux NutriSciences, Resana, Italy, Test Report: 18/000458102, date of issue 22/10/2018, E.D.T.A. 17% LOT 18010 EXP. 01.2023). Conversely, Tetraclean NA [64], a patented citric acid-based chelator for smear layer removal, was later reported as an irritant because of its low pH, and is thus not recommended for clinical use (Analytical Report: AAA86523, Eurofins Number: STULV18AA0037-1) by Eurofins Biolab Srl (Vimodrone, Milano, Italy).

From what has been highlighted above, therefore, when creating a new root canal irrigating agent, we must consider the irritating power and its pH rather as well as its cytotoxicity. As observed previously by M.T.T. assay results, both BioAKT citric acid-based complex increased cell viability significantly at 0.5% concentration (Table 1). Recently, BioAKT has shown to have antimicrobial activity on dentin discs from human teeth contaminated with *E. faecalis* biofilm [23]. Conversely, data available concerning the concentration of the various chemicals constituting BioAKT Endo root canal irrigant, the instructions for its clinical use, the safety data sheet, as well as its antibacterial activity are lacking. Because of this lack of information on its antimicrobial effects, in the present study, the antibacterial action of this new class of irrigants was evaluated on *E. faecalis* in laboratory settings. In line with the data available in the literature [23], BioAKT confirmed a remarkable effect in treating both planktonic and sessile *E. faecalis* in ‘in vitro’ experiments. However, such results were almost indistinguishable from the BioAKT Endo. Despite the promising antibacterial properties herein found, these new root canal disinfectants suffer from some critical issues such as their toxicity related to low pH, which could limit their safe clinical use [57]. To solve this problem, the lowest concentration of these classes of irrigants that can accomplish the cleanness of the root canal system should be used clinically. It must be considered that when an acidic solution is diluted with water, the concentration of hydrogen ions (H^+^) decreases and the pH of the solution increases towards a neutral value [65]. It is essential to underscore that both 0.5% silver citrate solutions (Table 1) correspond to a 1:200 diluted chemicals with a pH value of 3.7 (measured by a pH meter G.L.P. 22 as previously described), higher than their initial concentration (pH 1.7). Our findings have found that at dilutions greater than 1:16, the silver citrate solutions had no antibacterial and antibiofilm activity (Table 4, Supplementary Materials Figures S3 and S4, Table S1) because, at lower concentrations and higher pH values, the solutions improve the survival of bacteria [66], underlining the evidence that these chemical alterations could affect the disinfection of the root canals. Conversely, in our laboratory investigation on dentinal wall samples irrigated with undiluted and diluted silver citrate solutions, the smear layer removal capability of the diluted solution was preserved similarly to that undiluted (Supplementary Materials, Figure S1). For the reasons set out, further studies should be conducted using this class of irrigants to determine the most appropriate dilution capable of guaranteeing biocompatibility and ensuring an effective root canal cleaning.

## 5. Conclusions

Within the limitations of the methodology used, it can be assumed that both silver citrate solutions were toxic when used at higher than 0.5% concentration. Both BioAKT and BioAKT Endo solutions had similar antimicrobial activity, chemical composition, and pH value in their original formula. No antimicrobial and antibiofilm activity was found when BioAKT Endo was diluted. For the first time, the smear layer removal ability of diluted silver citrate complex was established, limitedly to the number of samples examined. Further in vivo studies are needed on this class of chemicals using safe concentrations to validate the outcome of the present study.

## Figures and Tables

**Table 1:** Mean values, standard deviations (S.D.), and 95% confidence intervals (CI) of mouse fibroblasts viability (based on M.T.T. assay) for the different concentrations of the two solutions. *p*-values of comparisons are also indicated (Mann–Whitney test).

Concentration	BioAKT	BioAKT Endo	*p*-Value
Control	1.29 ± 0.04 (1.22, 1.35)	1.25 ± 0.12 (1.06, 1.45)	0.89
0.25%	0.98 ± 0.04 (0.91, 1.05)	1.05 ± 0.07 (0.95, 1.16)	0.34
0.5%	0.95 ± 0.07 (0.85, 1.06)	0.95 ± 0.07 (0.83, 1.06)	1.00
1.0%	0.54 ± 0.06 (0.43, 0.64)	0.53 ± 0.02 (0.49, 0.57)	1.00
2.5%	0.067 ± 0.002 (0.06, 0.08)	0.070 ± 0.004 (0.06, 0.08)	0.20
5.0%	0.072 ± 0.001 (0.070, 0.074)	0.077 ± 0.006 (0.068, 0.086	0.11

**Table 2:** Results of Tukey’s test for mouse fibroblasts viability with BioAKT

Tukey’s Multiple Comparison Test	Mean Diff,	q	Significance	95% CI of Diff
0.25% vs. CONTROL	−0.3060	13.74	***	−0.4061 to −0.2059
0.5% vs. CONTROL	−0.3352	15.06	***	−0.4353 to −0.2352
1% vs. CONTROL	−0.7495	33.66	***	−0.8496 to −0.6494
2.5% vs. CONTROL	−1.219	54.76	***	−1.319 to −1.119
5% vs. CONTROL	−1.214	54.53	***	−1.314 to −1.114
0.25% vs. 0.5%	0.02924	1.313	ns	−0.07083 to 0.1293
0.25% vs. 1%	0.4435	19.92	***	0.3434 to 0.5436
0.25% vs. 2.5%	0.9131	41.01	***	0.8130 to 1.013
0.25% vs. 5%	0.9080	40.79	***	0.8080 to 1.008
0.5% vs. 1%	0.4142	18.61	***	0.3142 to 0.5143
0.5% vs. 2.5%	0.8838	39.70	***	0.7838 to 0.9839
0.5% vs. 5%	0.8788	39.47	***	0.7787 to 0.9789
1% vs. 2.5%	0.4696	21.09	***	0.3695 to 0.5697
1% vs. 5%	0.4646	20.87	***	0.3645 to 0.5646
2.5% vs. 5%	−0.005032	0.2260	ns	−0.1051 to 0.09504

ns = not significant; *** = *p* < 0.0001.

**Table 3:** Results of Tukey’s test for mouse fibroblasts viability with BioAKT endo

Tukey’s Multiple Comparison Test	Mean Diff,	q	Significance	95% CI of Diff
0.25% vs. CONTROL	−0.2003	6.165	**	−0.3463 to −0.05426
0.5% vs. CONTROL	−0.3029	9.324	***	−0.4490 to −0.1569
1% vs. CONTROL	−0.7252	22.32	***	−0.8712 to −0.5791
2.5% vs. CONTROL	−1.183	36.40	***	−1.329 to −1.037
5% vs. CONTROL	−1.175	36.18	***	−1.321 to −1.029
0.25% vs. 0.5%	0.1027	3.160	ns	−0.04339 to 0.2487
0.25% vs. 1%	0.5249	16.16	***	0.3788 to 0.6709
0.25% vs. 2.5%	0.9825	30.24	***	0.8364 to 1.129
0.25% vs. 5%	0.9751	30.01	***	0.8290 to 1.121
0.5% vs. 1%	0.4222	13.00	***	0.2762 to 0.5683
0.5% vs. 2.5%	0.8798	27.08	***	0.7338 to 1.026
0.5% vs. 5%	0.8724	26.85	***	0.7264 to 1.018
1% vs. 2.5%	0.4576	14.08	***	0.3115 to 0.6036
1% vs. 5%	0.4502	13.86	***	0.3042 to 0.5962
2.5% vs. 5%	−0.007384	0.2273	ns	−0.1534 to 0.1387

ns = not significant; ** = *p* < 0.01; *** = *p* < 0.0001.

**Table 4:** Minimum inhibitory concentration (M.I.C.), minimum bactericidal concentration (M.B.C.), and minimum biofilm eradication concentrations (M.B.E.C.) of the tested solutions

Solutions	Dilution			
	MIC	MBC	MBEC 1 min	M.B.E.C. 3 min
BioAKT	1:16	1:8	1:8	1:16
BioAKT Endo	1:16	1:8	1:8	1:16

**Table 5:** Effect of the tested solutions on dead cell ratio (C.L.S.M. assay)

Solutions	1 min	3 min
Control vs. Bioakt	ns	ns
Control vs. BioAKT Endo	ns	*
Bioakt vs. BioAKT Endo	ns	ns

* *p* < 0.05.

## Supplementary Materials

The following are available online at https://www.mdpi.com/1996-1944/13/21/5019/s1, Figure S1: Representative scanning electron microscopy micrographs of root canal walls after final rinse with distilled water, BioAkt Endo, BioAkt Endo 0.5%. A uniform coating of small spherulites and thick deposits of aggregated spherulites were observed on the surface of samples treated with BioAkt Endo. Original magnification 3000×, 6000×; Figure S2: Evaluation of the chemical composition (spectra) and the element distribution (elemental mapping) of samples treated with distilled water (Control), BioAkt Endo, and BioAkt Endo 0.5% were conducted with energy-dispersive X-ray spectroscopy (E.D.X.). The samples examination confirmed that the micro-agglomerations on the root dentin treated with BioAkt Endo were principally constituted of silver (Ag), calcium (Ca), and phosphorous (P). The percentages of silver (Ag), calcium (Ca), and phosphorous (P) in BioAkt Endo were higher when compared to control and 0.5% formula. E.D.X. no detected silver in BioAkt Endo diluted at 0.5%; Figure S3: C.L.S.M. 3D reconstruction of *E. faecalis* biofilm after different treatments. The bactericidal effect of BioAKT Endo 0.5% (ratio of live/dead cells) was comparable to saline compared to BioAKT Endo in its original formula; Figure S4: Biofilm removal efficacy of the tested solutions on pre-formed *E. faecalis* biofilm analyzed by C.L.S.M. Residual biomass is represented in percentage concerning the positive control treated with only saline. Whole bars represent the total biomass; the green fraction is viable cells, in red are depicted the dead cells still encased in the biofilm. Similarly to saline (C+), BioAKT Endo diluted at 0.5% do not show antibiofilm and antibacterial activity; Table S1: Statistical significance of pre-formed *E. faecalis* biofilm treatment analyzed by C.L.S.M. performed employing a one-way analysis of variance (ANOVA) followed by Bonferroni’s Multiple Comparison Test.
