# Supplementary material for: Evaluation of Cytotoxicity and Antibacterial Activity of a New Class of Silver Citrate-Based Compounds as Endodontic Irrigants
Source: Materials (Basel). 2020 Nov 6;13(21):5019. doi: 10.3390/ma13215019 (PMC7664443; doi:10.3390/ma13215019)
Supplement: Supplementary file 1 [file materials-13-05019-s001.pdf]

materials

Supporting Information

Evaluation of Cytotoxicity and Antibacterial Activity of a New Class of Compounds Silver Citrate-Based as Endodontic Irrigants

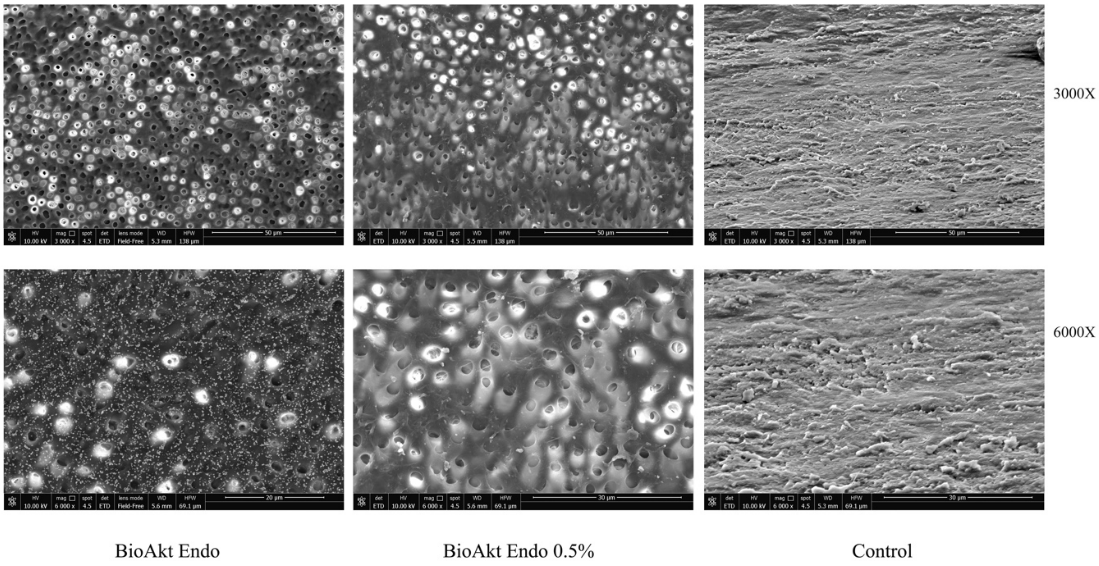

Figure S1. Representative Scanning Electron Microscopy micrographs of root canal walls after final rinse with distilled water, BioAkt Endo, BioAkt Endo 0.5%. A uniform coating of small spherulites and thick deposits of aggregated spherulites were observed on the surface of samples treated with BioAkt Endo. Original magnification 3000×, 6000×.

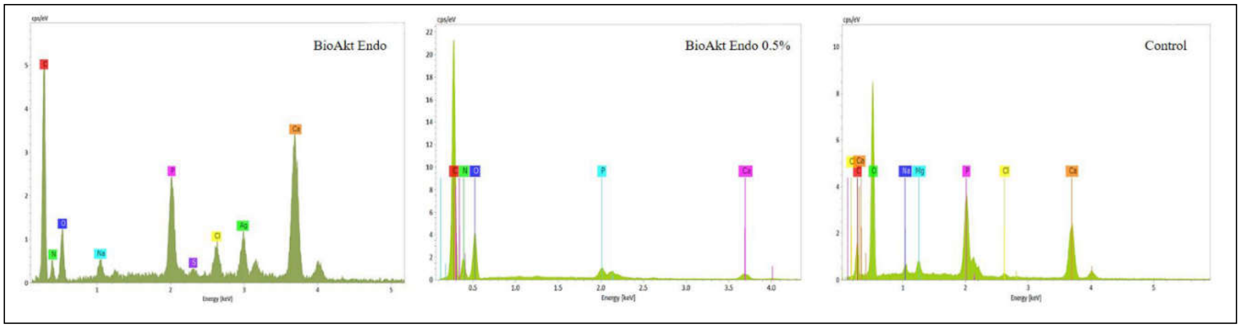

Figure S2. Evaluation of the chemical composition (spectra) and the element distribution (elemental mapping) of samples treated with distilled water (Control), BioAkt Endo, and BioAkt Endo 0.5% were conducted with energy-dispersive X-ray spectroscopy (EDX). The samples examination confirmed that the micro-agglomerations on the root dentin treated with BioAkt Endo were principally constituted of silver (Ag), calcium (Ca), and phosphorous (P). The percentages of silver (Ag), calcium (Ca), and phosphorous (P) in BioAkt Endo were higher when compared to control and 0.5% formula. EDX no detected silver in BioAkt Endo diluted at 0.5%.

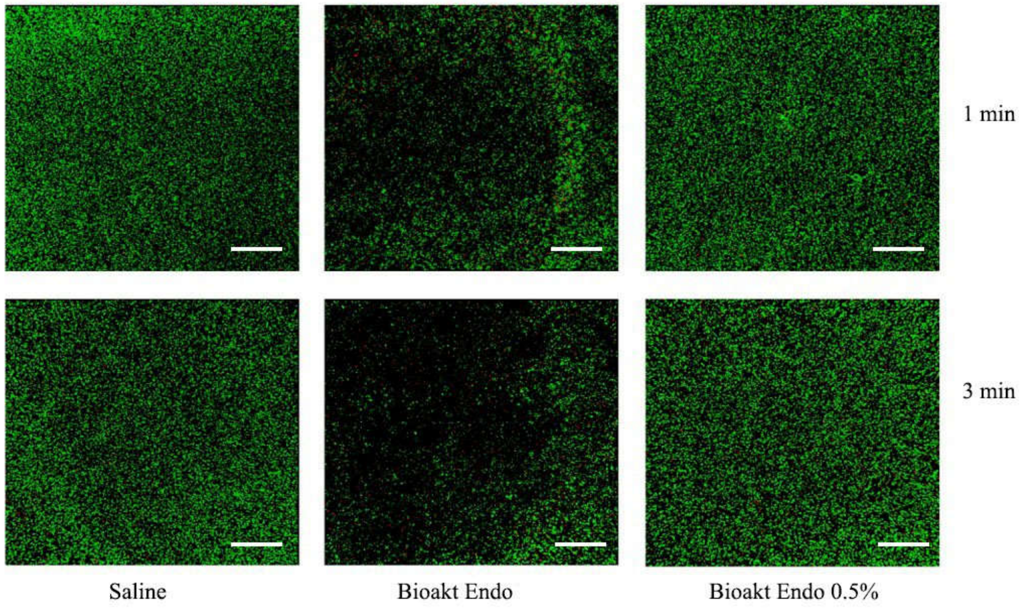

Figure S3. CLSM 3D reconstruction of *E. faecalis* biofilm after different treatments. The bactericidal effect of BioAKT Endo 0.5% (ratio of live/dead cells) was comparable to saline compared to BioAKT Endo in its original formula. Scale bar: 100μm

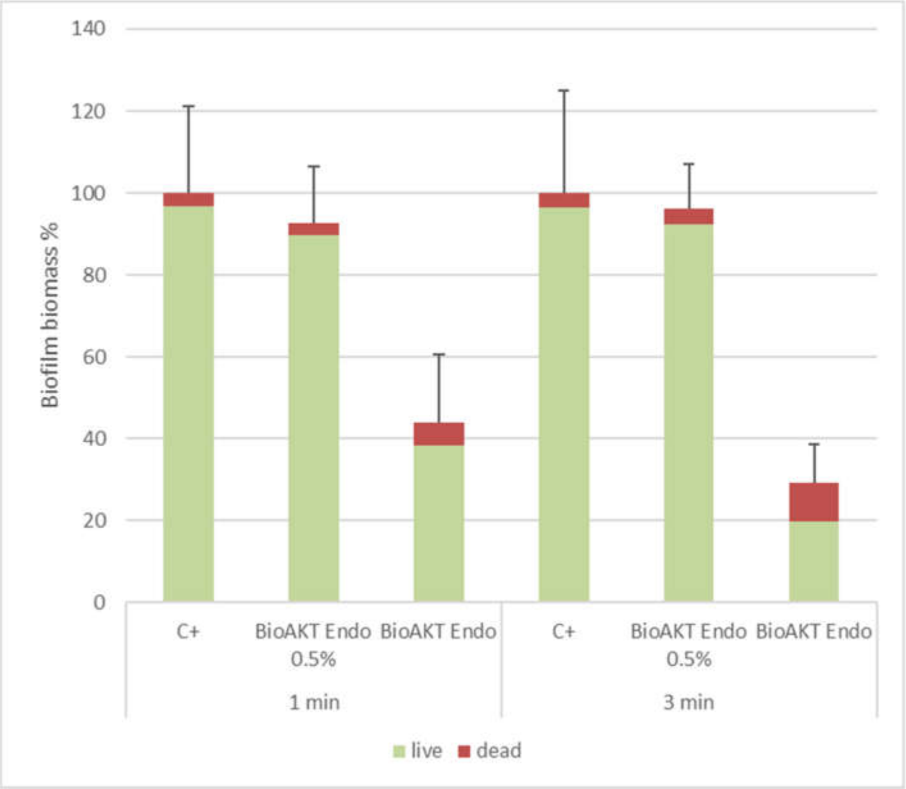

Figure S4. Biofilm removal efficacy of the tested solutions on pre-formed *E. faecalis* biofilm analyzed by CLSM. Residual biomass is represented in percentage concerning the positive control treated with only saline. Whole bars represent the total biomass; the green fraction is viable cells, in red are depicted the dead cells still encased in the biofilm. Similarly, to saline (C+), BioAKT Endo diluted at 0.5% do not show antibiofilm and antibacterial activity.

Table S1. Statistical significance of pre-formed *E. faecalis* biofilm treatment analyzed by CLSM performed employing a one-way analysis of variance (ANOVA) followed by Bonferroni's Multiple Comparison Test.

	**Bonferroni's Multiple Comparison Test**	**Significance**	
		**1 min**	**3 min**
Biomass	C+ vs BioAKT Endo 0.5%	ns	ns
	C+ vs BioAKT Endo	*	**
	BioAKT Endo 0.5% vs BioAKT Endo	*	**
Dead cells	C+ vs BioAKT Endo 0.5%	ns	ns
	C+ vs BioAKT Endo	ns	*
	BioAKT Endo 0.5% vs BioAKT Endo	ns	*

ns = not significant; * $p < 0.05$, ** $p < 0.01$.
